# A Call for Culturally Responsive Transformational Leadership in College Sport: An Anti-ism Approach for Achieving Equity and Inclusion

**DOI:** 10.3389/fsoc.2020.00065

**Published:** 2020-08-28

**Authors:** Joseph N. Cooper, Ajhanai C. I. Newton, Max Klein, Shannon Jolly

**Affiliations:** ^1^Leadership in Education, University of Massachusetts Boston, Boston, MA, United States; ^2^Leadership in Education, University of Connecticut, Mansfield, CT, United States; ^3^Department of Kinesiology, University of Georgia, Athens, GA, United States

**Keywords:** anti-racism, anti-sexism, culturally responsive leadership, college sport, equity

## Abstract

A longstanding fact in college sports in the United States (U.S.) is the reality that inequities, inequalities, and discrimination have been major issues preventing institutions from fostering harmonious diversity and inclusion. Several reasons for these persistent outcomes include the prevalence of implicit bias, homologous reproduction, hegemonic, and toxic masculinity/patriarchy, colorblind racism, and abstract liberalism to name a few. In addition, multi-level factors (macro-, meso-, and micro-) also influence the existence and salience of negative organizational cultures and climates particularly for groups that are underrepresented and marginalized in society. Despite the fact several leadership styles have been enacted and numerous policy reforms have been adopted over the years, inequities in representation, occupational mobility, position retention, and quality of experiences persist along racial and gender lines. As a result, the purpose of this manuscript is to offer innovative transformational leadership approaches that incorporate anti-racism, anti-sexism, and culturally responsive stances toward achieving true equity and inclusiveness in sport. Using interdisciplinary theories such as the anti-racism framework and culturally responsive leadership, this manuscript presents a paradigm shift for college sport leadership with the intent of cultivating paramount experiences for people across diverse backgrounds.

## Introduction

On Saturday, March 24, 2018, Felisha Leggette-Jack, Head Coach of the University of Buffalo Bulls basketball team, reflected on her professional journey as an African American woman during a National Collegiate Athletic Association (NCAA) Tournament post-game press conference (Mandell, 2018). In response to a question about the state of diversity in college sport leadership in the United States (U.S.), Leggette-Jack offered the following poignant indictment on the current state of the NCAA and its member institutions in regards to their commitment to diversity, inclusion, and equity: “I'm saddened by it. I understand the problem. I know that the majority of women basketball players look like me. I think these young women, if we really care about them as people, that they will have role models that look like them…” (Mandell, 2018, p. 1). Her *personal troubles* and experiences with racism and sexism in sport as an aspiring coach are emblematic of broader *public issues* in a society and industry that have failed to implement culturally inclusive policies and practices (Cooper, 2012). The current status quo and scholarly research indicates many racial minorities and women in these positions have a desire and ideas on how to improve diversity and inclusion, but too often they are not afforded the opportunity to lead or initiate the efforts to implement these changes (Singer and Cunningham, 2012, 2018). Moreover, in 2018, African American women only constituted 12% of Division I head women's basketball coaches while simultaneously constituting 43% of the women's basketball student-athletes (NCAA, 2019a). Alarmingly, this disparity was nearly identical a decade prior in 2008, when African American women accounted for only 10% of Division I head women's basketball coaching positions and 46% of the women's basketball student-athletes (NCAA, 2019a). *The Institute for Diversity and Ethics in Sport* (TIDES), the leading source on diversity in sports reporting in the U.S., issued college sport a C+ grade for 2018 (Lapchick et al., 2019b). According to the NCAA, their inclusion statement expresses a commitment to diversity, inclusion and gender equity among participants within their association (e.g., college athletes, coaches, and administrators) (NCAA, 2019b). More specific to inclusion, the NCAA highlights the following core areas: (a) disability, (b) international, (c) lesbian, gay, bisexual, transsexual, and questioning (LGBTA), (d) race/ethnicity, and (e) women (NCAA, 2019b). Yet, the aforementioned trends are prevalent across multiple NCAA sports (37) and across all three divisions (I, II, and III) despite the fact that the association hired its first Chief Inclusion Officer in 2003 (the late Bernard Franklin) (Rietmann, 2017) and restructured its Office of Inclusion in 2010 (NCAA, 2020a).

Representation is one of the many prevailing issues in college sport that result in persisting inequities, inequalities, and discrimination outcomes at NCAA member institutions. Since the inception of the NCAA in 1906, racist and sexist beliefs were embedded in the organizational policies and identity (Cooper et al., 2017). Concomitantly, women were not perceived as qualified leaders in social and political spheres and did not legally secure their voting rights until 1920 (Burton and Leberman, 2017). Historically, African Americans were largely excluded from athletic participation at historically White institutions (HWIs) (Cooper, 2019). In terms of leadership, African Americans were deemed as inferior intellectually and thus unfit to be leaders in all facets of society including sport (Cooper, 2019). Given the historical, political, social, and cultural factors surrounding the establishment of the NCAA, the modern day racial and gender disparities in leadership roles reflects a perpetuation of its racist and sexist origins. We also acknowledge there a range of discriminatory policies and practices grounded in heterosexism, ableism, and numerous marginalized identities [see Cunningham (2019) for an expansive analysis on these topics]. However, for the purpose of this manuscript we are focusing on race and gender. Hence, without strategic counter-hegemonic disruption, racialized and gendered hierarchies will continue to prevail.

Although, representation in leadership positions is vitally important and receives the most public attention (e.g., TIDES racial and gender report cards, NCAA inclusion initiatives, etc.), it only constitutes one aspect of the broader problem of racism and sexism in college sport. The entrenched nature of the NCAA and its member institutions reflects how they exist as racialized and gendered organizations. Ray (2019) explained how racialized organizations “…inhibit agency, legitimate the unequal distribution of resources, treat Whiteness as a credential, and decouple organizational procedures in ways that typically advantage dominant racial groups” (p. 46). More specifically, the author asserted that the interplay between the racial superstructure of the dominant racial ideology (Whiteness is superior), racial substructure of schemas (binaries, emotions, and implicit), and the racial structure of rules (formal and informal) and resources (material and social) result in sustained stratification of power [see Table 1 in Ray (2019), p. 33]. Within the context of the NCAA, the embedded nature of Whiteness along with the schema of Black marginalization, exploitation, and neglect has resulted in colorblind racist rules and resources (Cooper et al., 2014, 2017). Evidence of the NCAA's racialized structure include the perpetual underrepresentation of Blacks in leadership positions, athletic exploitation via the overrepresentation of Blacks in high-profile revenue generating sports of football and men's basketball, academic exploitation via consistent graduation gaps between Blacks and Whites, and the lack of adequate addressing of socioemotional challenges facing Black college athletes at HWIs (Cooper et al., 2017). Moreover, the unjust enrichment of White college sport stakeholders (e.g., NCAA headquarters, corporate sponsors, conference commissioners, HWI athletics departments, and predominantly White Olympic sports) and unjust impoverishment of Black college athletes and HBCUs signifies the racialized organizational structure of the NCAA (Hawkins, 2010; Cooper et al., 2014; Feagin et al., 2015). In addition, the array restrictive NCAA bylaws that control college athletes' time, ability to engage in educationally purposeful activities, freedom to interact with agents and related union representatives, and overall rights all reflect how components of the racialized organization (Ray, 2019).

Related to gender, Acker (1990) argued that organizations are not gender-neutral, but rather androcentric. The author specified how sexism is reflected in organizations via gender segregation of work, income and status inequality along gender lines, cultural images of androcentrism are invented and reproduced, and individual gender identity as products of organizational processes and pressures. In terms of segregation of work, the creation of the Senior Women's Administrator (SWA) position and the nebulous responsibilities associated with this role as well as the lack of a male designated equivalent underscore how these women are subjugated within NCAA athletic departments (Grappendorf et al., 2008; Hoffman, 2010; Schneider et al., 2010). Regarding income and status inequality, Osborne and Yarbrough (2000) highlighted how women earned sixty-two percent of what men were awarded in intercollegiate athletic positions. Despite performing similar duties, women in coaching and administrators continue to experience labor exploitation and gender discrimination. Related to cultural images of androcentrism, on the corporate partner Turner Sports Network website, the promotion of the NCAA's March Madness is populated with images of male coaches of men's and teams and in fact a picture of a women's head coach can only be located once an ancillary tab is selected (NCAA, 2020b). The subordination of women in gendered organizations is not coincidental, but rather it is “an integral part of those processes” that govern androcentric organizations such as the NCAA (Acker, 1990). In a survey of athletic directors across all three NCAA divisions (I, II, III), Whisenant et al. (2002) found that “…men have institutionalized their control over the most senior levels of management within intercollegiate athletics” (p. 489). A key finding from their study revealed that women have less representation at the Division I level, the highest revenue generating level, compared to Divisions II and III (Whisenant et al., 2002). The impact of gender stereotypes upon women leaders in sport has created a “leaky” pipeline of career accession for female associate athletic directors (Hancock and Hums, 2016), which has continued to perpetuate gendered organizations (Acker, 1990).

In spite of the NCAA's shortcomings on diversity and inclusion aims, it is worth noting that they have not been silent on key equity issues over the past few years (Table 1). Since the early 1990s, the NCAA has engaged in a range of efforts to improve diversity and inclusion within their association. The efforts include, but not limited to, the creation of a Gender Task Force in 1993, the sponsoring of annual Equity and Inclusion forums dating back to 2010, and the presentation of awards for diversity and inclusion to member institution athletic departments dating back to 2013 to name a few (NCAA, 2019a). In 2016, the NCAA created a *Presidential Pledge and Commitment to Promoting Diversity and Gender Equity in Intercollegiate Athletics* (NCAA, 2020c). The pledge reads as follows:

*Consistent with our mission and values, our institution, a member of the National Collegiate Athletic Association, pledges to specifically commit to establishing initiatives for achieving ethnic and racial diversity, gender equity and inclusion, with a focus and emphasis on hiring practices in intercollegiate athletics, to reflect the diversity of our membership and our nation*.
*We recognize and value the experiences individuals from diverse backgrounds bring to intercollegiate athletics. To that end, we will strive to identify, recruit and interview individuals from diverse backgrounds in an effort to increase their representation and retention as commissioners, athletics directors, coaches and other leaders in athletics. As part of this commitment, we will also engage in a regular diversity, inclusion and equity review to inform campus policy and diversity initiatives*.
*We understand this to be a collective responsibility we owe to student-athletes, staff, our athletics programs and the entire campus community* (NCAA, 2020c, p. 1).

**Table 1:** Examples of diversity and inclusion initiatives within division I athletic departments^a^.

**Institution**	**Initiative(s)**	**Support**	**Program description**	**Outcomes**	**Limitations**
University of Oregon	Community and inclusion committee	Oregon athletics department	Develops athletics strategic action plan for diversity. Reviews D&I policies and conducts implements D&I trainings and hiring practices	Increase in percentage of minority (POC, women, LGBTQ+) employers. Created a women's support group that increased women employers and cultivate safe environment for women in athletics	While the outcomes are published broadly, there are no publicly accessible measurable outcomes of these initiatives
Pennsylvania State University	Intercollegiate athletics diversity council	Athletics and institutionally supported	Provides a curriculum and strategic plan that fosters and educates staff on D&I practices.	Increase in women and POC in leadership positions. Ally training that supports an accepting environment for LGBTQ+ persons.	There are no publicly accessible measurable outcomes of council initiatives
University of Texas-Austin	Diversity and inclusion council	Athletics and institutionally supported	Reviews policies and develops strategic plans to increase D&I strategies for staff, leadership and athletes. Partners with Office of Inclusion and Equity to increase cultural competence of athletics org	Ally training to develop a welcoming staff for LGBTQ+ employees. Increase in employees who are female and Black or Hispanic.	There are no annual publicly accessible outcomes of the initiatives (most recently published outcomes were 2017).
University of Nebraska-Lincoln	D&I staff lunch and learn D&I summit UNL out and allied	Athletics and institutionally supported	Devotes time and resources to spread awareness and educate staff members on the importance of D&I (race, gender, sexual orientation) within the organization.	An increase in employees who are diverse (race, gender, sexual orientation). A safe space for women and LGBTQ+ individuals.	Though acknowledged by the NCAA for their diversity efforts, there is no publicly accessible data to support the broad positive outcomes of the D&I programing.
University of Wisconsin-River Falls	Falcons united	Athletics and institutionally supported	Develops D&I strategies to address social justice, race, privilege, LGBTQ+	Staff participate in D&I summits to improve race relations and safe spaces for employees	There are no publicly accessible measurable outcomes of council initiatives
Bowling Green University	We are one team	Athletics and institutionally supported	Uses sport to promote friendship between stereotyped groups in student athletes, staff and leadership.	Increase in diversity initiatives and policies. Received NCAA Minority Opportunities Athletic Association's Award for D&I.	There are no publicly accessible measurable outcomes of council initiatives

a *The organizations highlighted above have received recognition from the NCAA for their commitment to diversity for coaches, staff, athletes and community at large. Most of these institutions have multiple initiatives within their D&I councils or committees that are not explicitly describing the outcomes of their programming*.

Despite the well-intentioned nature of this pledge, there are several issues. First, a glaring omission from the pledge is an explicit acknowledgment of how previous and current policies and practices are responsible for the racial and gender inequities that persist (Cooper et al., 2017; Cunningham, 2019). In today's climate, given the passage of Title VII and Title IX and a host of other Civil Rights and anti-discrimination laws, the tone of this pledge is nominal. In other words, this pledge essentially states the NCAA will seek to be in compliance with federal laws and mainstream liberal views that suggest people from diverse backgrounds deserve equal opportunities for employment in the U.S. There is no direct confrontation toward or radical resistance against systemic racism and sexism (Ray, 2019). Rather the pledge appears to be a politically correct statement of intentionality to foster diversity and inclusion across the NCAA and its member institutions (Cooper et al., 2017). However, in order to address the issues associated with the deeply rooted oppressive ideologies such as racism and sexism, a more confrontational and courageous leadership approach is necessary.

The NCAA *Presidential Pledge* also primarily focuses on surface-level diversity in the form of *representation* as opposed to deep-level diversity in the form of *transforming the culture* (e.g., environment, ways-of-thinking, ways-of-doing, innovative strategies, culturally inclusive philosophies, etc.) of college sport when instituted with critical consciousness and culturally responsive (Scott, 2014; Cooper et al., 2017). Although, equity evasive efforts can manipulate both surface- and deep-level diversity to augment inequities (Hoffman, 2010), an important point by critical organizational change scholars is being intentional about identifying systemic issues and creating policies, processes, and evaluation metrics that assess multi-level outcomes regarding equity (Acker, 1990; Cunningham, 2019; Ray, 2019). On the NCAA's Inclusion Office website (NCAA, 2019a), regular reviews of campus policy and diversity initiatives are highlighted, and this strategy is duly noted. Notwithstanding, these efforts and messaging do not communicate what occurs after these reviews are complete and who is involved in them. Without transparency, in conjunction with the recent TIDES (Lapchick et al., 2019a) and NCAA (2019b) demographic data reports, it is clear structural change has yet to manifest through current efforts.

Another limitation of this pledge is the lack of enforcement for non-agreement or compliance (Lapchick et al., 2019a). Supporting institutions, conferences, and other entities can sign the pledge expressing their agreement with its sentiments, but there is no accountability for abiding by it or penalties for institutions who decline to sign it and uphold its sentiments. Conversely, the NCAA has only recently proposed bylaws to remove restriction on college athletes ability to profit from their own image and likeness, penalize college athletes for receiving arbitrary “impermissible” benefits, restricts coaches' behavior within and beyond athletic spaces, and a plethora of other constraints that are intended to supposedly *uphold the values of integrity, ethics, and sportspersonship*. Yet, when it comes to holding institutions accountable for *creating and sustaining racially and gender equitable and inclusive environments*, the NCAA remains passive and engages in more symbolic gestures instead of implementing more substantive culturally responsive efforts. Hence, the purpose of this manuscript is to offer innovative transformational leadership approaches that incorporate an anti-racism and anti-sexism stance toward achieving true equity and inclusion in college sport. For the purposes of the current analysis, we define equity as the fair treatment of all people with attention toward redressing social injustices against specific groups such as racial minorities^1^ and women both acutely and longitudinally.

## Research on Barriers and Reasons for Racial and Gender Inequities in College Sport

Our social identities not only impact how individuals navigate our unique social world, but how they navigate the workplace and professional domains (Cunningham, 2019). Thus, disparate organizational treatment based on race and gender are often reflective of pathological biases embedded in society (Wells and Kerwin, 2017). In order to grapple with why issues of racism and sexism remain in college sport organizations, we must understand its history, which is rooted in Whiteness and maleness (Fink et al., 2001; Hextrum, 2020). As stated earlier, the NCAA did not emerge with a consideration of racial minorities and women. As such, this historical precedent explains the creation of barriers for women and racial minorities as well as why college sports fail to perpetuate abstract liberalism (Bonilla-Silva, 2017; Cooper et al., 2017). Bonilla-Silva (2017) defines abstract liberalism as “…using ideas associated with political liberalism (e.g., “equal opportunity,” the idea that force should not be used to achieve social policy) and economic liberalism (e.g., choice, individualism) in an *abstract* manner to explain racial matters” (p. 28). Thus, the absence of anti-racism and anti-sexism rhetoric from NCAA policies and practices underscore the salience of abstract liberalism.

## Barriers Facing Women

Over the past several decades, there has been an abundance of scholarly literature focused on identifying and challenging the barriers facing women seeking sport leadership positions. For example, Burton (2015) conducted a meta-analysis of sport management literature that examined the underrepresentation of women in sport leadership positions and found barriers were present at a micro- (interpersonal), meso- (institutional), and macro-levels (association-wide and societal). These findings underscore the systemic nature of sexism (implicit and explicit) facing aspiring women sport leaders. Along the same lines, Sartore and Cunningham (2007) argued the dominant gender ideology and accompanying gender-role meanings associated with sport are the primary restrictions placed upon women seeking leadership positions. The authors posited that societal trends (also referred to as *macro processes*), historically and contemporarily, can have adverse effects on women whereby they unintentionally internalize self-limiting beliefs about their capacities to fulfill leadership positions in sport (referred to as *micro processes*) (Sartore and Cunningham, 2007).

Moreover, Burton et al. (2009) highlighted how social stereotypes of gender impact how certain college sport leadership positions are conceptualized and eventually fulfilled. The authors assessed how specific college sport leadership positions (e.g., athletic director, compliance coordinator, and life skills coordinator) have assumed gender characteristics that are either masculine or feminine. Even though, there were no significant differences for the athletic director position related to socially constructed feminine characteristics, men disproportionately fulfill these roles (Burton et al., 2009). This level of sexism is a vivid depiction of organizational biases fueling sport scholars to claim that sexism and gender inequity in sport is not a coincidence, but rather institutionalized (Cunningham, 2008; Walker and Sartore-Baldwin, 2013; Fink, 2016). Prominent barriers impacting women in college sport leadership are issues of gender stereotyping particularly the assumption of being detrimentally motherly and emotional (Bower et al., 2015), lacking experienceoverseeing football and men's basketball programs and operations (Yiamouyiannis and Osborne, 2012; Taylor and Hardin, 2016), and a dearth of female mentorship (Taylor and Hardin, 2016; Wells and Kerwin, 2017).

The issue of mentorship has often been associated with women being excluded from the old boys' club, a network of predominantly White men disseminating resources and opportunities to other White men, perpetuating homologous reproduction of this group as leaders (Shaw, 2006; Walker and Bopp, 2011; Walker and Sartore-Baldwin, 2013; Singer and Cunningham, 2018; Lapchick et al., 2019b). For example, Katz et al. (2018) found that the SWA network is less cohesive compared to male-dominated athletic director (ADs) and consequently the appointment of an SWA was found not to be the most successful path to AD ascension because these women were “detached from the center of the network as only a single female AD” (McDowell and Cunningham, 2009; Katz et al., 2018, p. 145). The aforementioned multi-level issues [micro- (interpersonal), meso- (institutional), and macro-levels (association-wide and societal)] grounded in sexism are also prevalent in the institutional racism within the NCAA, which underscores the interconnectedness between oppressive ideologies.

## Barriers Facing Racial Minorities

Sport leadership scholars have documented the adverse impact of occupational segregation in athletic departments (McDowell et al., 2009; Cunningham, 2010; Burton and Leberman, 2017; McDowell and Carter-Francique, 2017). The phenomenon of occupational segregation has an adverse impact upon women and racial minorities, but when examining specific historically marginalized communities, Blacks experience distinctive challenges. Utilizing a case study methodology, McDowell et al. (2009) found that African American administrators cited structural barriers related to discrimination encounters, lack of access to social networks, and lack of opportunities via hiring practices as reasons for the underrepresentation of racial minorities in senior level leadership positions. Such a discriminatory perspective coupled with systemic barriers of racism such as a lack of access to pipeline mentorship networks contributes to only 20 Black ADs across all three NCAA divisions when HBCUs are excluded (Singer and Cunningham, 2018). Relatedly, Blacks are overrepresented in academic support roles compared to other leadership positions such as athletic director, associate athletic director, and head coach (McDowell and Cunningham, 2009; McDowell et al., 2009). A Black male participant in Wells and Kerwin (2017) study expressed how he felt racial minorities are perceived to be “doers” and are overlooked as managers (p. 136).

In an effort to understand the interconnectedness between social systems, Cunningham (2010) applied a multi-level analysis of the factors that contribute to the underrepresentation of African American coaches. The macro-level factors included institutional practices, political climate, and stakeholder expectations. Two influential stakeholder groups in college sport are alumni and donors. According to Cunningham (2010), it is not uncommon for these stakeholders to engage in aversive racism. The author offered the following definition of aversive racists:

…people who consciously and sincerely support egalitarian ideals and do not believe that they personally harbor prejudiced feelings toward racial minorities; nonetheless, these persons unconsciously have feelings of unease toward historically disadvantaged groups and therefore seek to avoid interracial interactions (Cunningham, 2010, p. 399).

We likened this type of racists to what Bonilla-Silva (2017) described as new racism or colorblind racism, which refers to the four frames of “*abstract liberalism, naturalization, cultural racism*, and *minimization of racism*” (p. 26). Among these four frames, the most germane to the current analysis is the abstract liberalism (Bonilla-Silva, 2017), which a concept that was created by Europeans and grounded in Euro-centric cultural values that are in direct contrast with African epistemologies, ontologies, and axiology (Nichols, 1974). Abstract liberalism purports individualism and meritocracy as keys to successful mobility in society without any consideration for how social structures and policies create racial and gender inequalities (Kendi, 2019). In addition, among these stakeholders are individuals connected to political structures at the local, state, and national level that adopt aversive racists postures, which further complicates the unfair opportunities for racial minorities to pursue college sport leadership positions.

The meso-level factors included prejudice and discrimination, exclusionary leadership prototypes, and organizational cultures of diversity. For example, McDowell and Carter-Francique (2017) documented how African American women are disadvantaged by leadership prototypes that are grounded in racist and sexist stereotypes as opposed to being assessed on their merit, talent, and ability. Moreover, Cunningham (2010) posited that prejudice increases when there is a lack of standardized procedures. In these instances, ambiguous and arbitrary decisions are issued, and these thought processes are often grounded in implicit biases that reinforce rather than resist the status quo and mainstream oppressive ideologies. The commonplace process of new White coaches being hired shortly after a previous one was fired illustrates the power of the good ol' boys network despite federal laws that are supposed to guarantee equity and non-discrimination in employment. Additionally, recruitment efforts for numerous athletic staff positions often involve word-of-mouth whereby exclusive networks have access to this information as opposed to using targeted approaches such as sending out vacancies via culturally diverse organizational listservs and/or to HBCUs or minority serving institutions (MSIs) (Cunningham and Sagas, 2008; Cooper et al., 2017).

The micro-level factors include head coaching expectations and intentions and occupational turnover intentions (Cunningham et al., 2001; Cunningham, 2010). Black coaches are less likely to be rehired once fired even when they have better or comparable win-loss records to their White peers, which reflects differential coaching expectations based on race (Lapchick et al., 2019a). Collectively these issues present both access and treatment discrimination for racial minorities (Greenhaus et al., 1990; Cunningham and Sagas, 2005). Access discrimination occurs when there are formal and informal barriers to entry into an occupational field whereas treatment discrimination occurs after a person is employed within an organization and there is a mismatch between espoused values of diversity and inclusion and actual practices of racism (formally and informally). Both access and treatment discrimination have contributed to a myriad of negative outcomes for racial minorities including low career satisfaction and mobility and declined health wellness (Cunningham et al., 2006). As such, the removal of barriers to diversity and inclusion could translate into a host of short-term and long-term organizational benefits.

## Benefits of Diversity

As the U.S. consistently becomes a more diverse country, many organizations are intentionally developing, organizing, and enhancing their diversity policies and practices to promote a positive environment of equity and inclusion (McPhillips, 2020). According to a U.S. News report, 70% of the largest metropolitan cities are more racially and ethnically diverse compared to 2010 (McPhillips, 2020). Along the same lines, an American Council on Education (ACE) report revealed that undergraduate students of Color constitute nearly half of all enrolled students (45.6%) in 2016, which was a nearly 17% point increase from 1996 (29.6%) (AAC U News, 2019). In a climate that pushes individuality, innovation, and creativity, diversity is critical to an organization's economic growth and success. Latimer (1998) surmised that diversity promotes creativity and problem-solving capabilities. In addition, diversity fosters a more elaborate knowledge base and an accumulation of perspectives that can positively influence decision-making (Duchek et al., 2019). Through the mixing of minds, a greater variety of perspectives call for a higher level of critical analysis that allows organizations to be more productive. More specifically, in the workplace, Jones and George (2009) identified several benefits of increasing diversity. One benefit involves accessing a *broader pool of applicants and talent* (domestically and internationally). The mixture of cultural backgrounds and experiences allows for companies to develop strategic partnerships with heterogeneous markets (Armache, 2012).

Another benefit is *increased retention* via allowing members of an organization to feel valued as integral contributors to the team. Burrell (2016) noted that strong diversity also reassures members that progression or growth within the organization is based purely on merit rather than demographics. A third benefit of diversity is the offering of *broader service range to customers*. According to Burrell (2016) diverse organizations are more equipped to connect with diverse consumers. A fourth benefit of diversity is the opportunity to create *more innovative and creative solutions*. An understanding of an individual's strengths, weaknesses, and methods to foster healthy relationships have been shown to enhance creativity, innovation and problem-solving capability (Bassett-Jones, 2005). *Increased workplace productivity and job performance* is yet another benefit of diversity. When diverse differences are viewed as assets and healthy collaboration is valued, overall productivity can increase within organizations (Cunningham, 2008, 2009, 2019). *Increased competitiveness and profitability* is another byproduct of organizational cultures of diversity, which contributes to increases in resource acquisition from employees and marketing through cultural sensitivity (Cox and Blake, 1991).

Diversity also offers numerous benefits within sport. Athletic organizations that adopt an inclusive environment with positive interracial interactions foster increased cognitive development and socialization among its members (Hirko, 2009). According to van Knippenberg et al. (2004), the *categorization-elaboration model* explains how diverse groups improve decision-making, information transmission, and problem-solving in organizational settings. Thus, a collaboration of people with unique backgrounds enables new perspectives to emerge and increases group/team performance. Building upon this framework, Cunningham and Melton (2011) found that diversity (including sexual orientation) contribute three positive outcomes in sport organizations: (a) *enhanced decision-making capabilities*, (b) *improved marketplace understanding*, and (c) *goodwill associated with social responsibility*. Proactive sport organizations adopt a broad view of diversity, which encompasses valuing inclusion in their policies, procedures, and leadership practices.

Despite research indicating the benefits of diversity, many sports organizations still reflect organizations of similarity and inequity (Fink et al., 2001; Cunningham, 2008). Fink et al. (2001) found that different diversity approaches in sport organizations result in varied outcomes. For example, *compliance strategies* associated with retaining talented workers, avoiding lawsuits, and having diverse consumers/fan bases do not have the same positive outcomes as *proactive strategies* that involve creating workplaces that attract talented employees, enhance employees' sense of value, engage employees in decision-making processes, and promote creative and innovative ideas for organizational change (Fink et al., 2001). Relatedly, Cunningham (2009) posited that sport organizational cultures of diversity compared to cultures of similarity are more likely to have diversity and inclusion initiatives embedded in their policies (e.g., zero tolerance policy for discrimination, mandatory diversity trainings, etc.) and practices (e.g., mentoring programs and proactive recruitment strategies for hiring candidates from underrepresented backgrounds). As sport organizations are multi-level systems, activities and diversity initiatives on the micro level (i.e., employees' sense of belonging) influences the meso-level (i.e., team bonding activities reflecting cultural diversity and broader social capital) and macro-levels (i.e., successful diversity policies and procedures) of the organization. Thus, in order to reap the ever-evolving benefits of diversity, sport organizations must incorporate a multi-level approach to diversity and inclusion.

## Leadership Styles in College Sport Research

Burton (2015) delineated three prominent leadership styles in college sport, which include transformational, transactional, and servant. The authors described transformational leadership as an approach that focuses on inspiring and motivating followers to maximize their personal growth for organizational success. Transformational leaders demonstrate charismatic traits and prioritize vision and goal setting for collective benefits (Bass, 1990; Bass and Riggio, 2006; Welty Peachey et al., 2014; Burton, 2015). In contrast, transactional leadership focuses on incentivizing follower behavior through exchange of benefits for performance outcomes (Burton, 2015). Transactional leadership is goal and objective centered whereby organizational productivity is paramount and individual's development is primarily considered in connection with broader business aims (Welty Peachey et al., 2014).

The predominant leadership style in Division I college sports in the U.S. is transactional (Burton, 2015). In concert with neoliberal capitalist societies and aims, transactional leadership styles lead to individualistic interests becoming prioritized rather than leaders considering the systemic oppression being imposed upon those they lead (i.e., African American women seeking leadership positions in sport—see McDowell and Carter-Francique (2017) for a detailed analysis). Hence, if people of African, Latino, Asian, and Indigenous descent and women seek to gain access to leadership positions and be retained in these roles, then they must assimilate to this transactional leadership approach. We argue college sports, as well as host of other industries, is best served with transformational leaders who adopt culturally responsive and multicultural orientations. In other words, people of oppressive racial groups (e.g., Blacks, Latinos, Asians, and Indigenous) and women should not be forced to adopt or assimilate into transactional leadership approaches in order to be viable candidates among the coaching and administrator ranks in college sport. Rather the system and structure of college sport should recognize the shortcomings of colorblind and de facto patriarchal transactional leadership and its role in creating persistent racial and gender inequities.

Another prominent leadership style across professional industries including sport is servant leadership. According to Burton et al. (2009), servant leadership refers to “the interaction between leader and follower and emphasizes how leaders can be attentive to the needs of followers, show concern for their followers, and nurture and emphasize the needs of followers” (p. 27). Servant leadership is particularly beneficial for organizations that are centralized and share a set of common core values (i.e., small private religious schools and their athletic departments). However, a limitation of servant leadership is its lack of applicability to larger heterogeneous groups where conflicting values and philosophies may exist among stakeholders. For example, in college sports, the fact that Whites constitute a majority of the leadership roles and college athlete population means there is limited representational power for people of Color. As such, a servant leadership approach in college sport would seek to address the needs of multiple groups that may have different (albeit at times overlapping) needs, preferences, and demands (e.g., differences in cultural backgrounds, political orientations, and racial histories and experiences in the U.S. and beyond). If a servant leader sought to meet the needs of a majority of their followers in this context, then inevitably certain groups whether underrepresented numerically or influence wise would be subordinated (either intentionally or unintentionally). Notwithstanding, it is worth noting that servant leadership under certain conditions could overlap with transformational leadership particularly if an organization espouses values of democratic (as opposed to autocratic) and collective leadership (Scott, 2014). Hence, we contend a shift toward a culturally responsive transformational leadership approach would not only reduce the current racial and gender inequities in college sport, but also lead to improved organizational outcomes related to performance, stakeholder satisfaction, and overall impact on society. Gay (2002) defined of culturally responsive teaching as “…using the cultural characteristics, experiences, and perspectives of ethnically diverse students as conduits for teaching them more effectively” (p. 106). We adapted this definition for the current analysis to refer to culturally responsive leadership as the intentional infusion of cultural characteristics, experiences, worldviews, and insights of ethnically diverse people as conduits for working with them more effectively as a transformational leader.

## Current Issues With Failed Leadership in College Sport

When NCAA member institutions do not have culturally responsive measures in crisis, their behavior is at a minimum questionable and at a maximum unethical and illegal (Roby, 2014; Smith and Willingham, 2015; Cooper et al., 2017). An example of failed leadership in college sport involved officials at Baylor University who did not appropriately handle numerous instances of rape and sexual assault within its football program. In 2014, former Baylor football players Tevin Elliot and Sam Ukwuachu faced sexual assault charges (Chavez and Croft, 2018). Elliot was found guilty and sentenced to 20 years in prison, whereas Ukwuachu was initially found guilty in 2015 before the conviction was overturned in 2018 and he was granted a new trial (Chavez and Croft, 2018; Lavigne, 2018a). Later in 2019, he was once again found guilty. In 2016 another Baylor football player, Shawn Oakman, was arrested on charges of sexual assault, but he was found not guilty in 2019 (Chavez and Croft, 2018; Witherspoon, 2019). In 2017, a former Baylor volleyball player filed a lawsuit against the university for mishandling her report of being gang-raped by eight Baylor football players in 2012. The lawsuit, which ended in a settlement, purported “that the university allowed a “rape culture” to persist within the football program” (Lavigne, 2018b, p. 1). In a 2017 lawsuit, it was alleged that from 2011 to 2014 at least 31 football players committed at least 52 rapes (Tracy and Barry, 2017). As a direct result of these numerous cases and the rape culture that existed at Baylor, head football coach Art Briles was fired and university president Ken Starr resigned (Kerner et al., 2017; Chavez and Croft, 2018). Additionally, Title IX Coordinator Patty Crawford resigned because reportedly the university did not provide her appropriate resources or allow her to do her job properly. An independent investigation, conducted by the law firm Pepper Hamilton, found that Baylor prioritized winning football games over the safety of its students (Meyer, 2017). The culture at Baylor represented a sexist transactional leadership approach that prioritized the financial impacts and institutional prestige of winning football games while simultaneously silencing victims/survivors of sexual assaults and limiting the effectiveness of the Title IX office.

Lastly, the case of Larry Nassar, a former doctor and osteopathic physician for the United States of America Gymnastics (USA Gymnastics) and Michigan State University (MSU) also reflected leadership failures and lack of cultural responsiveness. Nassar was accused of sexually abusing 265 young women and girls dating back to 1992 (Connor, 2018). MSU reported the first complaint against Nassar was received in 2014, yet he was allowed to remain with the university and treat patients under specific restrictions (Connor, 2016). Nassar was eventually fired for violating this agreement in 2016 after allegations of sexual assault against him were reported by *The Indianapolis Star* (Connor, 2016). Similar to the PSU case, several key officials including MSU gymnastics coach Kathie Klages was aware of sexual abuse allegations against Nassar as early as 1997 and accused of dismissing sexual abuse complaints against Nassar and pressuring survivors to stay silent (Finley, 2017).

Along with Nassar, William Strampel, the former dean of the College of Osteopathic Medicine at MSU, was arrested and charged with criminal sexual conduct for allegedly groping a student, storing nude photos on his computer, and possessing a video purported to be a training video of the pelvic floor manipulation procedure created by Nassar (Joseph and del Valle, 2019). The last two significant MSU figures that notably failed to act were former president Lou Anna Simon and former athletic director Mark Hollis. After pressure from the Michigan House of Representatives, the MSU Board of Trustees pushed Simon to resign (Tracy, 2018). In an attempt to repair the damage caused by their failure to lead, the MSU Board of Trustees voted to establish a $10 million fund to reimburse Nassar's victims for counseling services (Kozlowski, 2018). However, survivors of Nassar's were not satisfied with this minimal effort by MSU and reached a settlement of $500 million with the university in 2018 (Kozlowski, 2018).

The aforementioned cases represent instances of failed leadership at NCAA member institutions. These are not the only cases of failed leadership among NCAA member institutions. However, these examples, as well as countless others including the Ohio State University wrestling sexual abuse case (Redden, 2020), the rape cases involving Vanderbilt University football players (Luther, 2015), and the University of Louisville men's basketball coaches using sex workers to recruit players (Barr, 2016) show that a lack of culturally responsive leadership is not unique, but pervasive in college athletics. Our operational definition for culturally responsive leadership is useful for understanding how these issues could have been mitigated or eliminated altogether.

The most significant theme that is present across the scandals at Baylor (Litman and Ruiz, 2016), MSU (Gibbs, 2018), and most within college sport (Harper and Donnor, 2017) is the cover up, or attempt to hide the scandal from becoming public. A culturally responsive approach would have prioritized not only those impacted by the failed leadership, but also recognized that the best course of action is to take responsibility, accept blame, and adjust policies and procedures to ensure the same issues cannot happen again. Furthermore, Harper and Donnor (2017) discuss how the overrepresentation of powerful White men in leadership positions considering the importance of female and Black athletes to an athletic department creates athletic departments with overly masculine and White cultural features that are inequitable and more susceptible to scandals. An athletic department that is culturally responsive would be less susceptible to scandals because the leadership is more evenly distributed across racial, ethnic, gender, and other groups. This more even distribution of leadership allows for more effective transformational leaders who are reflective of the makeup of an organization. As a result, we argue that a culturally responsive anti-racism and anti-sexism transformational leadership approaches prior to, during, and after crises would mitigate these issues of failed leadership in college sport.

## Anti-Racism (and Anti-Sexism) Philosophical Stance

In concert with previous critics of the NCAA (Bimper and Harrison, 2017; Cooper et al., 2017), we assert the current colorblind abstract liberalist approach to policy creation and implementation in U.S. college sports is a primary reason for the perpetual racial and gender inequities in leadership. In contrast to this passive approach, we call for a courageous and innovative approach to sport leadership that is grounded in anti-racism and anti-sexism. In his critically acclaimed book titled, *How to be an Anti-Racist*, African American historian Kendi (2019) presented a framework for countering racist policies and outcomes in society. According to Kendi (2019) a racist as “one who is supporting a racist policy through their actions or inaction or expressing a racist idea” (p. 13). In contrast, an anti-racist is “one who is supporting an antiracist policy through their actions or expressing an anti-racist idea” (p. 13). In relation to the NCAA, the absence of a *Rooney Rule* or *Eddie Robinson* type policy (New, 2016) and concurrent underrepresentation of racial minorities in leadership positions particularly in sports such as football and men's basketball (the highest revenue generating sports for the association) reflects the impact of *de facto* racist policies at the association and institutional levels (Cooper et al., 2017). Kendi (2019) outlined the distinction between racist framing and anti-racist framing:

One either believes problems are rooted in groups of people, as a racist, or locates the roots of problems in power and policies as an anti-racist. One either allows racial inequities to persevere, as a racist, or confronts racial inequities, as an anti-racist (p. 9).

The blatant disregard for how formal policies and informal practices create the perpetual inequities among leadership positions in college sport is an example of problematic liberalist orientations and according to Kendi (2019) by default this type of leadership is racist. In concert with previous anti-racist college sport scholarship, we posit the current racial inequities and inequalities in college sport are byproduct of *de facto* racist policies that have roots dating back to the establishment of the NCAA in the early 1900's (Cooper et al., 2017). Until these historical realities are acknowledged and confronted, racial justice will not manifest.

Expanding on this framework, we surmise the same argument applies to the differences between sexist and anti-sexist approaches. Institutional sexism is reflected in the fact that according to the most recent TIDES report on Division I Football Bowl Series (FBS) college sport racial and gender report, the institutions at this level of college sport received an F for gender hiring (Lapchick et al., 2019a). Leadership positions examined in this report included university presidents, conference commissioners, athletic directors, and faculty athletic representatives. Scholars have documented the range of reasons associated with the ideology of sexism that explain (not excuse) these trends (Walker and Bopp, 2011; Burton, 2015; Burton and Leberman, 2017; McDowell and Carter-Francique, 2017). Furthermore, the prevalence of sexual discrimination, assault, and abuse in college sports also illustrates the pervasive nature of sexism in this sector. We assert that current gender inequities in NCAA leadership ranks and throughout the organization are not a byproduct of biological sex or socially constructed gender differences, but rather a result of sexist policies and practices that disadvantage women particularly those from underrepresented and marginalized racial and sexual identity backgrounds.

The NCAA's passive and minimally progressive stance on persistent inequities among its leadership ranks across multiple levels prevents the association and its member institutions from adequately redressing the core issues of systemic racism and sexism. In order to address these issues, we must first recognize discriminatory treatment and outcomes exist *as a result of racist and sexist policies and practices* as well as a lack of anti-racist and anti-sexist policies. This position is akin to liberation sociology, which refers to “alleviating or eliminating various social oppressions and with creating societies that are more just and egalitarian…It adopts what Gideon Sjoberg has called a *countersystem* approach. A *countersystem* analyst consciously tries to step outside her or his own society to better view and critically assess it” (Feagin et al., 2015, p. 1). As Kendi (2019) noted, the opposite of racism is anti-racism not non-racism. Using an anti-racist and anti-sexist framework, our aim is to offer innovative culturally responsive leadership approaches that redress persistent racial and gender issues in college sport.

## Culturally Responsive Leadership

It has been well-documented how the NCAA was founded upon racist and sexist principles whereby racial minorities and women were largely excluded from leadership roles during the early twentieth century (Cooper et al., 2017; Katz et al., 2018). In recent years, several scholars have promoted culturally responsive leadership as a solution to the problems of racial inequities within K-20 schooling systems (Gay, 2002; Johnson, 2007). Given the fact that NCAA members are higher education institutions, we surmise a culturally responsive leadership approach would enhance the NCAA's efforts in redressing their longstanding racial and gender inequities. Building on the literature on culturally responsive pedagogy (Gay, 2002) and multicultural education in K-12 schooling (Ladson-Billings, 1994), Johnson (2007) examined the strategies of three urban school principal case studies where positive school-community-home relationships were cultivated with diverse populations. Each principal was involved with the International Successful School Principalship Project (ISSPP). An aim of the ISSPP was to foster culturally responsive leadership approaches that “affirm students' home cultures, increase parent and community involvement in poor and culturally diverse neighborhoods, and advocate for change in the larger society” (Johnson, 2007, p. 49). The author noted how the creation of this project was birthed from parents' feelings of marginalization and neglect in terms of school leadership. An example of how the principals affirmed students' home cultures was by working with their parents to address safety issues in the neighborhood. Rather than simply focusing on classroom instruction and behavioral expectations, the principals acknowledged the lived experiences and concerns of the students and their families beyond the school building. Similarly, Gay (2002) highlighted how storytelling, autobiographical case studies, scenarios (grounded in lived experiences as opposed to stereotypes), and vignettes are beneficial strategies associated with culturally responsive engagement.

As it relates to college sport, literature on racial minorities and women echoes these sentiments whereby their perspectives, unique experiences, and efforts have resulted in little structural change in leadership roles and policy development (Burton, 2015; Cooper et al., 2017; McDowell and Carter-Francique, 2017; Singer and Cunningham, 2018). Johnson (2007) defined culturally responsive leadership practices as “those that help to empower diverse groups of parents and make the school curriculum more multicultural” (p. 50). Similar to culturally responsive teaching (Villegas and Lucas, 2012), culturally responsive leadership involves *sociopolitical consciousness, cultural empathy*, and *a collective responsibility and commitment to inclusive learning and progress* (Johnson, 2007). Culturally responsive leadership is inherently critical of social inequalities and inequities and stimulates change through both policies (i.e., mandates) and practices (i.e., relationship building). Culturally relevant teaching focuses on “self and other, social relations, and knowledge” (Ladson-Billings, 1995, p. 483) whereas culturally responsive leadership “…requires a more thorough knowledge of the specific cultures of different ethnic groups, how they affect learning behaviors, and how classroom interactions and instruction can be changed to embrace these differences” (Gay, 2002, p. 114). We argue college sport leaders have utilized culturally biased transactional approaches that prioritize status quo sustainment (read: Whiteness and maleness) and corporate business aims rather than adopting culturally responsive transformational leadership policies and practices centered on equity and social justice. As noted earlier, the NCAA through its Office of Inclusion and *Presidential Pledge* express their collective responsibility and commitment to diversity and inclusion, but they fail to demonstrate sociopolitical consciousness and cultural empathy in policy or practice.

In concert with Johnson (2007) and Kendi (2019), we assert a precondition of culturally responsive transformational leadership is a level of compassion for those who are most disadvantaged by systemic racism and sexism [among several^2^ isms such as classism, ageism, ableism, heterosexism, colorism, etc.—see Cunningham (2019) for an in-depth overview of these isms]. This compassion must not remain in an awareness state, but rather progress to sustained concerted action. Culturally responsive leadership also incorporates the “history, values, and cultural knowledge” of leaders from underrepresented and marginalized backgrounds (Johnson, 2007, p. 51). Enhanced cross-cultural communication is vital for culturally responsive leadership. For example, understanding the importance of communalism, ethic of care, and reciprocity are core values among the African American community (as well as numerous Diasporas). A useful strategy for creating positive cross-cultural communication is role playing scenarios whereby staff are required to read a narrative of historically marginalized person and enact this role. These narratives could be derived from scholarly literature on these groups or anecdotes from mass media. In these activities, White males could role play a Black person (male, female, or non-gender conforming), female (across any racial group aside from White), or non-gender conforming person and be confronted the reality (albeit role playing) with how discrimination based on race and gender feels. In addition, assigning staff readings of and small group discussions on texts such as Kendi (2019), DiAngelo (2018), and additional authors of historically marginalized backgrounds could be helpful in enhanced cross-cultural awareness and empathy. These discussions and activities could conclude with policy reform action items. Thus, promoting values of individualism, competition, and conflict are inherently divergent from the cultural orientation of African Americans. Thus, within many NCAA athletic departments, the values promulgated do not align with those who have been historically excluded and marginalized and hence assimilation is the only route to acceptance (read: partial inclusion), which does not translate into full inclusion. The problem with this arrangement is it may achieve surface-level diversity, but it does not reflect and in fact it dismisses and prevents the presence of deep-level diversity which would result in culturally inclusive and transformative environments.

## Application of Culturally Responsive Transformational Leadership Approaches in College Sport

Building on the works of sport leadership scholars who have called for more inclusive policies and practices at the intercollegiate level (Walker and Bopp, 2011; Melton and Cunningham, 2012; Burton and Leberman, 2017; Cooper et al., 2017; McDowell and Carter-Francique, 2017; Cunningham, 2019), we assert the missing links from current NCAA leadership are deep-level diversity, culturally responsive approaches, and a commitment to anti-racist and anti-sexist strategies. An important first step in anti-ism approaches is the explicit recognition of the historical oppression, inequities, and inequalities that exist as a result of detrimental ideologies such as White racism, sexism, and capitalism. In sport, the ubiquitous promotion and acceptance of colorblindness, ahistoricism, purported neutrality and objectivity, and the myth of meritocracy have resulted in inadequate approaches to achieve full diversity and inclusion [see Ray (2019) for a more expansive discussion on racialized organizations]. Creating anti-racist and anti-sexist policies is not about privileging one group over another, but rather it is about redressing historical and systemic oppressions and leveling the occupational playing field. U.S. society and its institutions were not founded on equality. Thus, anti-ism culturally responsive transformational leadership is seeking to accomplish and create an environment that has not previously existed in the U.S. broadly. Recognizing the magnitude of this feat, we must courageously champion true equity, diversity, inclusion, and justice, which involves a comprehensive understanding of sociohistorical, sociopolitical, socioeconomic, and sociocultural developments and their concomitant impact on structural arrangements and personal experiences. Hence, in the spirit of anti-ism approaches, we outline specific recommendations to advance efforts toward achieving true diversity, equity, and inclusion in college sport in the U.S.

In an effort to combat access discrimination on the basis of race and gender, a recommendation that many businesses have already started to incorporate into their organizational structures is establishing of committees that consist of internal and external advocates (i.e., the Organizing Committees for the Olympic Games to ensure racial and gender equity for its athletes, coaches, and fans) (Olympic Games, 2020). These organizing committees or councils act as a proactive approach to end current or prevent future inequity issues (Diversity Best Practices, 2019). Beyond merely creating these committees, we recommend they are afforded organizational power to enact and monitor the implementation of their recommendations. For example, the current NCAA Office of Inclusion could partner with groups such as National Association of Collegiate Women Athletics Administrators (NACWAA), National Association for the Advancement of Colored People (NAACP), National Urban League (NUL), Mentors in Violence Prevention (MVP), Black Lives Matter (BLM), and similar organizations (e.g., academic departments, cultural centers, and community organizations dedicated to racial and gender equity) to create policies, practices, and methods of evaluation to reduce and eliminate systemic racism and sexism and improve diversity and inclusion throughout the association and its member institutions. These entities reflect what Ray (2019) described as external sources of organizational racialization. Culturally responsive leadership requires increased sociopolitical consciousness and cultural empathy (Johnson, 2007) and thus these tenets could be infused into NCAA core values, policies, and practices. These strategic partnerships could enhance NCAA leadership accountability.

It is worth noting and commending the NCAA for their creation of the Dr. Charles Whitcomb Leadership Institute (NCAA, 2020b). This initiative is designed to improve ethnic minority representation and ascension in college sport leadership positions. The core purpose of this program strives to redress past and present racial disparities and the efforts are duly noted. Nonetheless, there are notable limitations and issues with this initiative. One limitation lies in the fact that the application process only accepts 18 candidates per cohort (NCAA, 2020b). In the spirit of anti-racism, we recommend the establishment of a broader leadership development effort whereby mentorships, internships, job shadowing, and related professional development activities in partnership with sport leadership undergraduate and graduate programs across the U.S. particularly at HBCUs, Minority Serving Institutions (MSIs), and HWIs that enroll racial minority students.

Although, the NCAA has created an Office of Inclusion and offered minority leadership trainings and mentorship programs, these meso- and micro-level efforts are not anti-racist and they are limited in effectiveness because they do not confront and transform current campus-wide and athletic department climates. For example, the Leadership Institute focuses on preparing racial minorities for careers in college sport leadership positions (NCAA, 2020b), but this framing positions them as the problem rather than the racist system that has disadvantaged them. Thus, educating and holding accountable White college sport leaders and changing policies to stimulate more diversity (surface-level and deep-level), equity, and inclusion would reflect an anti-racist approach. Providing more opportunities for racial minorities and women in environments that are grounded in racism and sexism does not address the core issues rather it creates the illusion of inclusion and allows for the victim-blaming neoliberal notions of representation to persist (i.e., White males are more represented in leadership roles because they are more fit to perform the duties). Cunningham (2010) purported that “.change efforts cannot focus on a single level, but instead, need to recognize and take into account the intersectionality of macro-, meso-, and micro-level factors” (p. 403). Transforming cultures through socialization and accountability/enforcement is vitally important for substantive change to manifest. Improving representation temporarily or removing access discrimination does not automatically eliminate treatment discrimination and thus a cultural shift grounded in equity and social justice is necessary. Focusing on individuals and groups without changing systems and cultures results in limited social change.

Moreover, an anti-racist and anti-sexist leadership approach would also include ongoing NCAA mandated multicultural, diversity discrimination, and culturally responsive leadership development educational programming grounded in anti-ism approaches. These efforts would reflect internal sources of organizational racialization (Ray, 2019). Multicultural competency trainings have been promoted in the past (Singer and Cunningham, 2018; NCAA, 2020a), but our recommendation differs because we call for anti-racist, anti-sexist, and anti-isms programming whereby participants are equipped with the skills necessary and expected to engage in counter-oppressive actions (Kendi, 2019). Using data including evocative quotes from scholarly literature to provide concrete examples of racism and sexism from first-hand accounts could be useful in education sessions. Beyond the education and programming, athletic departments could be held accountable for implementing on-going strategies to improve racial and gender equity at all levels of their organization (e.g., leadership, staff, and college athletes). For example, the Office of Inclusion disseminates educational resources, implements diversity centric programs and initiatives, and provides awards and scholarships to deserving athletic staff and college athletes in an effort to foster equity and equality. Creating recognition platforms at comparable levels as NCAA championship events is recommended to signal the prioritization of championing diversity, inclusion, equity, and social justice through sport leadership.

Regarding improving surface-level diversity via policy, anti-racist and anti-sexist policies would incorporate inclusion hires (currently practiced in the entertainment industry) akin to original intent of affirmative action laws to ensure system wide changes are imminent as opposed to wishful. This approach would involve position descriptions to be revised to indicate the valuing of multicultural skill sets and talents. In concert with culturally responsive leadership (Johnson, 2007), these positions would infuse the history, values, and cultural knowledge of diverse groups as opposed to being written from a traditionally privileged viewpoint (i.e., White male). The intentionality of updating position descriptions would increase the likelihood of a diverse talent pool and alter the current evaluation standards for people who occupy these positions whereby engaging in equity minded efforts would be valued more than colorblind or abstract liberalist actions. These position descriptions could also be reviewed in advance to posting by social justice and diversity oriented third-party entities. Furthermore, once these positions are fulfilled by individuals from underrepresented and marginalized backgrounds, the NCAA should recognize institutions that engage in progressive equity efforts grounded in the perspectives of these professionals. Personal biographies and philosophies at the leadership levels must reflect the diversity of the constituents who are served (i.e., college athletes).

Relatedly, anti-racist and anti-sexist leadership approach promotes data-driven policy and practice reform. Throughout this manuscript and current Special Issue, numerous research articles, books, and reports have outlined the multi-level issues contributing to the racial and gender inequities in college sport. The creation of the Office of Inclusion was in part a result of the awareness of this research. However, the lack of comprehensive and radical policy reform indicate there remains a disconnect between the magnitude and seriousness of the inequities that persist and the NCAA and its member institutions' urgency to change policies, practices, and cultures to be more inclusive. As such, we recommend the NCAA and member institutions use current and future research findings as a guideline for creating best practices. Outlining specific barriers noted in the research and creating specific action plans to redress these issues short-term and long-term is recommended.

Similar to the previous recommendation about recognition, the celebration of the headquarters and member institutions who consistently and effectively improve their organizations based on these standards (surface-level and more importantly deep-level diversity) would enhance diversity, equity, and inclusion efforts. Another anti-racist approach to diversity is the removal of the commonplace term “of Color” to reference multiple racial groups and rather specifically acknowledge distinct barriers for different racial groups such as Blacks. When the term “of Color” is avowed, the unique oppression of distinct marginalized communities is not disaggregated and their unique oppression becomes assumed with other marginalized communities (Cooper, 2016; Lu and Newton, 2019). For example, the *TIDES* report has been beneficial in highlighting racial and gender disparities in sport, but in several instances during its initial iterations the aggregation of racial groups via the term of Color assumed each racial group faced the same challenges, which is inaccurate and problematic (Cooper, 2016; Lu and Newton, 2019). Thus, it is important for anti-racist organizations to be intentional about acknowledging the uniqueness of each group's racialized oppression. It is also vital for organizations to acknowledge that language influences cognition and behaviors of employees. Policies that encourage employees to refrain from gendered language are recommended to eliminate gender-biases, as it contributes to sexist culture by making gender salient, and treating gender as a binary category, which can cause gender stereotyping and prejudice (Bigler and Leaper, 2015). To make the gender of employees salient, the usage of neutral terms over masculine/feminine forms should be utilized for titles, positions and addressing a group (e.g., Councilor vs. Councilman or councilwoman, Good afternoon everyone vs. Good afternoon ladies and gentlemen, etc.).

Along the same lines, an anti-racist approach would also acknowledge systemic anti-Black racism in college sports and adopt specific initiatives to increase Black representation in leadership positions particularly in sports where they are overrepresented among the college athlete demographic (Lapchick et al., 2019a). Cunningham (2010) reflected this approach when highlighting the statistic that African Americans were 7.6 times more likely be a football player than a head coach. One way in which diversity is measured is through the federal affirmative action approach whereby the percentage of racial or gender group within organizational leadership should reflect the percentages of these groups in the U.S. population (Cunningham, 2010). Another approach to measuring diversity in sport suggests the ratio should involve the proportion of former athletes compared to the proportion of those racial groups represented in coaching positions (Cunningham and Sagas, 2005; Cunningham, 2019). We recommend using this type of data whereby the percentage of players (current and former) is contrasted with the percentage of racial groups represented in leadership ranks as one standard for assessing anti-racist progress. In sports where certain racial groups are significantly underrepresented, the initial anti-racist aim would be to implement policies aimed at increasing the rate of participation and leadership in these sports to at least meet the percentage of these racial groups as represented in the general U.S. population, which would be akin to affirmative action standards (Cunningham, 2010). Once this goal is met, then more progressive diversity efforts can be pursued. The status quo of racial disproportionality and underrepresentation across and within sports (as athletes and in administrative leadership roles) should be viewed and treated as a problem that requires concerted action in both the short-term and long-term.

As it relates to the failed leadership examples highlighted earlier in the manuscript, we offer the following recommendations for how a culturally responsive, anti-racist, anti-sexist, and transformational leadership approach could have been implemented to redress these issues. Using a culturally responsive framework in the PSU case requires situating the context of the child sex abuse scandal whereby the news media coverage focused on the ethical failure of individuals as opposed the *larger cultural context of sport which normalizes toxic masculinity and the suppression of empathy via the celebration of violence and aggression particularly from male offenders* (Cooky, 2012). With regards to the Baylor cases, under a culturally responsive and anti-sexist transformational leadership approach, Baylor would not have ignored any sexual assault survivors, but rather officials would have given their Title IX office more power as well as fired the head coach who enabled this rape culture (whether intentionally or unintentionally) when the university first received reports (O'Neill, 2018). In addition, a culturally responsive anti-sexist transformational leadership approach would embed anti-sexism throughout the campus culture via policies and practices that communicate a zero tolerance for sexual assault and abuse. Strict penalty enforcement for violators of behavioral standards and the empowerment of survivors of and activists for sexual assault prevention are among the strategies that could adopted. The most promising, and culturally responsive, outcome from the scandal at Baylor is that the state of Texas adopted new legislation “that is more survivor-centered” (O'Neill, 2018, p. 214). Related to the MSU case, a culturally responsive and anti-sexist approach would involve the enforcement of policies and procedures that protect all individuals from abuse. While there were policies, procedures, and additional codes of conduct in place that Nassar ignored (Mountjoy, 2019), MSU additionally ignored Title IX policies and procedures in place to protect victims who come forward (Kitchener, 2018). Culturally responsive anti-sexist transformational leaders establish trust and demonstrate compassion with victims/survivors of abuse (Barr and Murphy, 2020). If these issues emerge, officials must accept culpability and engage in corrective action to create change immediately (preferably proactively, but if not feasible then swiftly after the issues are brought to light) (Frederick et al., 2019). Our recommendations are in concert with Mountjoy (2019) who stated that “safe sport can only occur if there is a change in the culture of sport to one where athletes are respected and empowered to speak and influence change” (p. 59).

Beyond the *Presidential Pledge* (NCAA, 2020c), the NCAA should have incentives and penalties in place for institutions that do not have or fail to implement diversity, equity, and inclusion action plans. Strategic partnerships should be encouraged and required for these action plans to increase the likelihood of their success. Similar to Title IX prongs, the NCAA could require institutions to demonstrate a history of improvement with regards to diversity and equity in leadership positions and organizational climate (i.e., as evidence of third-party quantitative and qualitative data collection and analysis), effective accommodation of underrepresented and/or marginalized groups, and/or fulfill a proportionality approach [as suggested by Cunningham (2010) and Lapchick et al. (2019a)]. Culturally responsive leadership would also involve redefining current success metrics for the NCAA as it relates to diversity, inclusion, and equity goals. More specifically, an anti-racist and anti-sexist agenda would measure success by through the reduction and elimination of racial and gender disparities in representation, retention, promotion, performance, and satisfaction. Another culturally responsive leadership recommendation is to include racial minority and women athletic directors, coaches, graduate assistants, and college athletes (current and former) in best practices discussions. Beyond nominated officials, we recommend including the voices of all athletic stakeholders via survey data, interviews, and focus groups. Gathering this type of national data across HWIs, HBCUs, and MSIs would enable the NCAA to create and implement more data-driven practices grounded in equity and cultural inclusion. Once this data is collected and analyzed by third-party researchers, policy and practice reforms can be adopted and monitored accordingly. Specifically, attributing reforms to data-driven approaches with diverse respondents would reflect culturally responsive leadership.

Moreover, an anti-racist approach does not view racism and capitalism as separate oppressive ideologies rather it acknowledges that exploitation through latter is inextricably linked to the former (Cooper et al., 2017). Engaging in anti-racist leadership means recognizing how mainstream ideologies such racism and capitalism work in tandem with social institutions to create adverse and inequitable experiences for historically marginalized groups and *more importantly engaging in anti-racist counter-actions against these systems and norms* (Kendi, 2019). For example, anti-racist leaders must acknowledge how the current NCAA model of amateurism is inherently exploitative of Black males (Hawkins, 2010). Black males are immensely overrepresented in football (55%) and basketball (56%) among Power five conference schools while concurrently only constituting 2.4% of overall undergraduate student enrollment at these institutions (Harper, 2018). Given the lucrative nature these two sports (Hawkins, 2010), an anti-racist leader would surmise that the current model of NCAA amateurism is grounded in capitalistic exploitation that intersects with racial oppression (Hawkins, 2010; Cooper et al., 2017; Cooper, 2019; Hextrum, 2020). In other words, Kendi (2019) described the intersection of these two oppressive forces when he said: “But it is impossible to know racism without understanding its intersection with capitalism” (p. 156). This understanding is timely, as the collegiate sport model is in a politicized juncture of scrutiny from politicians and U.S. state legislatures reforming their state amateurism laws (Tynes, 2019).

The collegiate sport model has long touted amateurism and abstract liberalism (Bonilla-Silva, 2017) in hopes of continuing to present collegiate sport as logical and ethical, whilst ignoring the racial inequity rife in the arrangement (Hawkins, 2010; Cooper et al., 2017). An anti-racist leader must remain attentive to how systemic oppressions (i.e., racism and capitalism) intersect to create unique experiences through policy creation and enforcement. An anti-racist leader must also develop an understanding and consciousness of how colorblind practices and language (Bimper and Harrison, 2017) and amateurism bylaws (Cooper et al., 2017) are influenced by capitalism and fuel racial injustice. Relatedly, Omi and Winant (2014) asserted that “drawing attention to race—racial identity and difference, racial inequality and oppression, racial exclusion and violence—allows us to question the inconsistencies and platitudes of colorblind racial ideology” extant in American society (p. 261). Meaning, an anti-racist leader cannot solely examine experiences of race devoid of considering how this social construct and identity intersects with other systems of oppression. As such, adopting race-based policies for the purpose of redressing past and current inequities is a reflection on anti-racist stance as opposed to the status quo colorblind racist approaches that view race-based approaches as inherently discriminatory against non-targeted groups. While steps have been made to address racial and gender shortcomings within sports organizations, it is imperative for leadership to implement proactive approaches to diversity. Another proactive approach is the emerging trend of diversity and inclusion administrators in athletic departments (Newton, 2019). These inaugural positions are theorized to increase in adoption (Newton, 2019) and their leadership should consider cultivating anti-racist and anti-sexist sport organizations.

## Conclusion

For the sake of fostering sport organizations of equity and inclusiveness, this manuscript calls for the adoption of an anti-racist and anti-sexist approach to diversity. Incorporating diversity policies and practices that address gender-biased language, overt and covert racial discrimination, and prioritize culturally responsive leadership strategies are vital to accomplishing deep-level diversity, equity, and inclusion in college sport. Overall, diversity has many benefits for sport organizations. On the micro-, meso- and macro- levels, promoting an organizational culture that fosters diversity, equity and inclusion increases productivity, loyalty and marketability for the organization as well as its stakeholders who are concerned with inclusive environments. Our work echoes the sentiments expressed by Cunningham (2010) when he stated the following ultimate goal of cultivating inclusive workplace environments in college sport:

Change is possible. But, it takes a collective effort—a unified endeavor to transform the institutionalized systems in place, ensure a political environment where diversity is valued, eradicate decision makers' prejudices, stereotypes, and discrimination, create and sustain university workplaces characterized by diversity and inclusion, and transform the coaching profession into one where opportunities for African Americans abound (p. 404).

In addition to African Americans, when all underrepresented and marginalized groups have access to leadership positions in college sport and subsequently receive equitable treatment for occupational mobility then success can be celebrated. Hence, it is our hope that culturally responsive transformational leadership grounded in anti-ism approaches is incorporated throughout college sport in the U.S. to redress past and current inequities and injustices, which could lead to the creation of more equitable, diverse, and inclusive spaces for all.

## Data Availability Statement

The raw data supporting the conclusions of this article will be made available by the authors, without undue reservation.

## Author Contributions

All authors listed have made a substantial, direct and intellectual contribution to the work, and approved it for publication.

## Conflict of Interest

The authors declare that the research was conducted in the absence of any commercial or financial relationships that could be construed as a potential conflict of interest.

## Footnotes

^1^We acknowledge the problematic nature of the usage of this term and later in the manuscript we articulate our stance against using terms such as “racial minorities” and “of Color” [see Cooper (2016) and Lu and Newton (2019) for a more in-depth analysis on this topic]. However, in an effort to be consistent with cited works throughout the manuscript we used this term to refer to racial groups that have been subjected to various and different types of systemic oppressions including, but not limited to, Blacks and African Americans, Hispanics and/or Latinas/os/x, Asians and Asian Americans, and Indigenous People.

^2^We recognize the breadth isms and the limitations of the current scope of analysis on racism and sexism. However, we feel given the extent of racism and sexism within college sport and its interconnectedness to related isms (e.g., colorism, heterosexism, etc.) that the title of the manuscript is appropriate.
